# Supplementary material for: The E3 ubiquitin ligase ZNRF2 is a substrate of mTORC1 and regulates its activation by amino acids
Source: eLife. 2016 Apr 22;5:e12278. doi: 10.7554/eLife.12278 (PMC4889327; doi:10.7554/eLife.12278)
Supplement: Supplementary file 1. — DOI: http://dx.doi.org/10.7554/eLife.12278.014 [file elife-12278-supp1.doc]

V-ATPase and Ragulator subunits identified in ZNRF2-GFP immunoprecipitates

| **UniProt ID** | **Mascot score** | **Peptides matched** | **Sequence coverage** | **Gene Symbol** |
| --- | --- | --- | --- | --- |
| Q93050 | 769 | 78 | 29% | ATP6V0A1 |
| Q9Y487 | 701 | 53 | 28% | ATP6V0A2 |
| P61421 | 458 | 44 | 25% | ATP6V0D1 |
| P38606 | 196 | 14 | 18% | ATP6V1A |
| P27449 | 159 | 14 | 11% | ATP6V0C |
| P36543 | 83 | 2 | 8% | ATP6V1E1 |
| Q96A05 | 46 | 3 | 8% | ATP6V1E2 |
| Q6IAA8 | 319 | 8 | 30% | p18 |
| Q9UHA4 | 86 | 3 | 8% | Mp1 |
| Q9Y2Q5 | 37 | 3 | 16% | p14 |
